# Prediction of the Hamstring Graft Size for ACL Reconstruction Using Different Axial Layers in Preoperative MRI

**DOI:** 10.3390/jpm14060582

**Published:** 2024-05-29

**Authors:** Moritz Florian Mayr, Markus Siegel, Elham Taghizadeh, Peter Obid, Hagen Schmal, Kaywan Izadpanah

**Affiliations:** 1 Department of Orthopedic Surgery and Traumatology, Freiburg University Hospital, Albert Ludwigs University Freiburg, Hugstetter Straße 55, 79106 Freiburg, Germany; markus.siegel@uniklinik-freiburg.de (M.S.); peter.obid@uniklinik-freiburg.de (P.O.); hagen.schmal@freenet.de (H.S.); kaywan.izadpanah@uniklinik-freiburg.de (K.I.); 2 Fraunhofer Institute for Digital Medicine MEVIS, Max-von-Laue-Str. 2, 28359 Bremen, Germany; elham.taghizadeh@mevis.fraunhofer.de; 3 Department of Orthopedic Surgery, University Hospital Odense, Sdr. Boulevard 29, 5000 Odense, Denmark

**Keywords:** ACL surgery, knee surgery, sports injuries, arthroscopy, individual surgery, hamstring, MRI, digital imaging

## Abstract

Background: In ACL reconstruction, it is desirable to assess preoperatively whether a sufficient graft diameter can be achieved with the planned tendon graft. The present study investigated the effect of the location of the cross-sectional area (CSA) measurement of the hamstring tendons in preoperative MRI on the correlation of the CSA with the intraoperative graft diameter. In addition, we analyzed whether the measurement results of examiners with different skill levels were comparable. Methods: A total of 32 subjects undergoing a single bundle ACL reconstruction using an autologous ipsilateral quadrupled hamstring graft (STGT) were included. The CSA of the semitendinosus and gracilis tendon was determined in preoperative MRI on six defined levels by three examiners. The intraclass correlation coefficient between the measurements of these observers was determined. The correlation between the sum of the CSA of both tendons (CSA STGT) and the graft diameter was investigated. Results: The interrater reliability was excellent on most of the investigated levels. A significant correlation between CSA STGT and the graft diameter was seen on all levels. The strongest correlation was found on the level 10 mm above the joint line. Conclusions: The measurement of the CSA STGT in the preoperative MRI 10 mm above the joint line enabled a good assessment of the achievable graft diameter in ACL reconstruction, independent of the examiners’ training level.

## 1. Introduction

Rupture of the ACL is a common injury in individuals doing sports, either professional or recreational. Due to an increase in physically active patients, the numbers are even on the rise. The autologous hamstring tendons are frequently used grafts for ACL reconstruction with low donor-site morbidity compared to other autografts [1,2]. Recent studies have shown a significant higher risk of rerupture if hamstring autografts with a diameter less than 8 mm are used [3,4,5]. However, the semitendinosus and gracilis tendons show a considerable interindividual variance [6,7,8]. Therefore, preoperative prediction of the hamstring graft size would be of considerable importance to offer an individualized surgical procedure for each patient. There are several studies that have investigated the correlation of radiological quantifiable parameters and the intraoperative diameter of a hamstring graft. Magnetic resonance imaging (MRI) measurements of the hamstring tendons’ cross-sectional area (CSA) show promising results. In the existing studies on this topic, different methods were used regarding the localization of the CSA measurement with divergent results. Often, only the data of a few selected MR scanners have been used [9,10,11,12,13,14,15,16]. This is in contrast with everyday clinical practice, where patients bring along their MRI data from different devices with varying quality into the orthopedic outpatient department, and the MR images are often evaluated by examiners with different skill levels. We hypothesize that the results of observers with different levels of expertise are comparable and that there is an effect of the location of the measurement on the correlation of the CSA with the intraoperative graft diameter. The aim of the present study was to investigate these two hypotheses.

## 2. Materials and Methods

**Figure 1:**
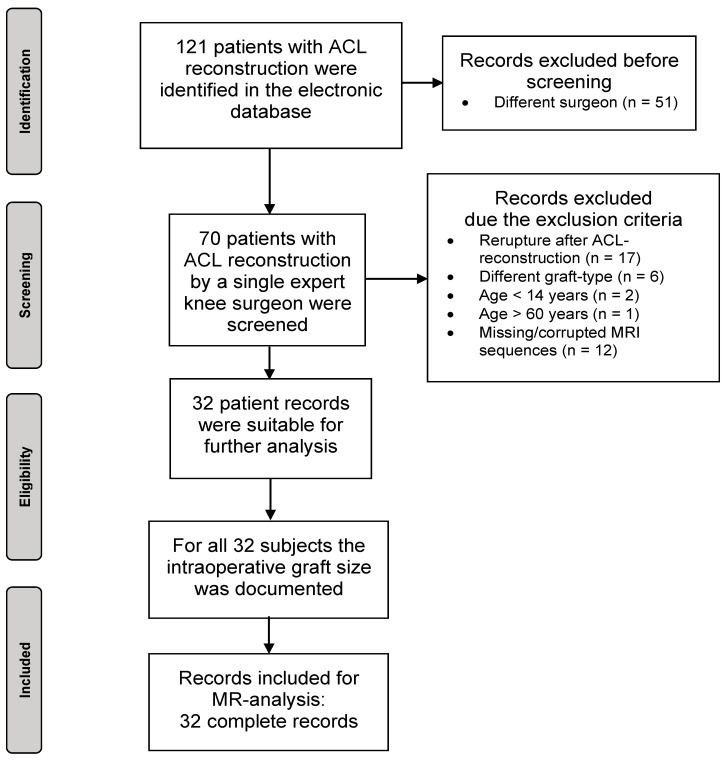
Flowchart of the selection process.

The present retrospective observational study included 32 patients who underwent a single bundle ACL reconstruction using an autologous ipsilateral quadrupled hamstring graft (semitendinosus tendon and gracilis tendon each doubled). The patients were identified from the electronic database of the clinical center retrospectively according to the ICD-code for ACL-rupture and only a single surgeon. The study was conducted in accordance with the Declaration of Helsinki. Approval was obtained from the institutional review board (447/19). The study was registered in the German Trials Register (DRKS 00031502). Inclusion criteria for patients consisted of an age between 14 and 60 years, ACL reconstruction with an autologous ipsilateral quadrupled hamstring graft (STGT), complete preoperative MRI dataset of the injured knee joint, and an intraoperative documented graft diameter. Patients with an age under 14 years and older than 60 years, previous ipsilateral ACL reconstruction, acute or chronic ipsilateral hamstring injury as well as missing or corrupted digital MRI data were excluded. A total of 121 patients with ACL rupture were identified. In seventy subjects, the ACL reconstruction was performed by a single expert knee surgeon and they were screened. Seventeen of these records had to be excluded due to a rerupture after ACL-reconstruction, six records because another graft-type was used, three records due to an age under 14 years or over 60 years, and twelve records because of missing or corrupted MRI sequences, resulting in thirty-two full records for further analysis (Figure 1).

### 2.1. Participants

Mean age was 27.8 ± 11.4 years, and 62.5% of the subjects were male. The anthropometric data of the subject pool are summarized in Table 1.

### 2.2. MRI Measurements

**Figure 2:**
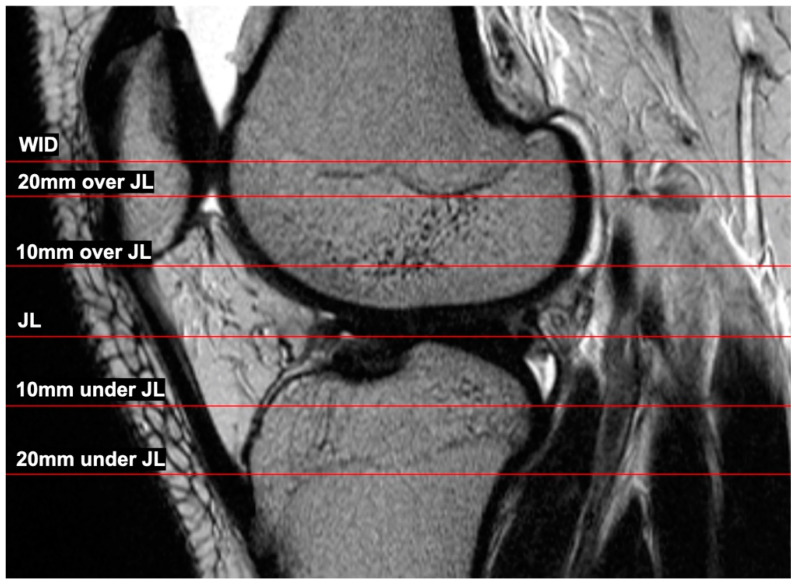
Sagittal presentation of the different levels where the CSA was measured in the axial layers.

The complete preoperative MRI dataset was extracted from the digital database of the clinical center. Both in-house and external MRI scans were used. The image data therefore came from a variety of MR scanners. For CSA measurement, the image processing program Horos (Version 3.2.1, Nimble Co LLC, Annapolis, MD, USA) was used. First, the joint line (JL) was determined in the sagittal plane. Afterward, axial layers 10 mm and 20 mm below and above the joint line as well as the axial layer at the level of the widest intercondylar dimension of the epicondyles were selected (Figure 2). Therefore, the following six axial slices were used for the CSA measurement:

- Level of the widest intercondylar dimension (WID);
- 20 mm over joint line (20 mm);
- 10 mm over joint line (10 mm);
- Joint line level (JL);
- 10 under joint line (−10 mm);
- 20 mm under joint line (−20 mm).

**Figure 3:**
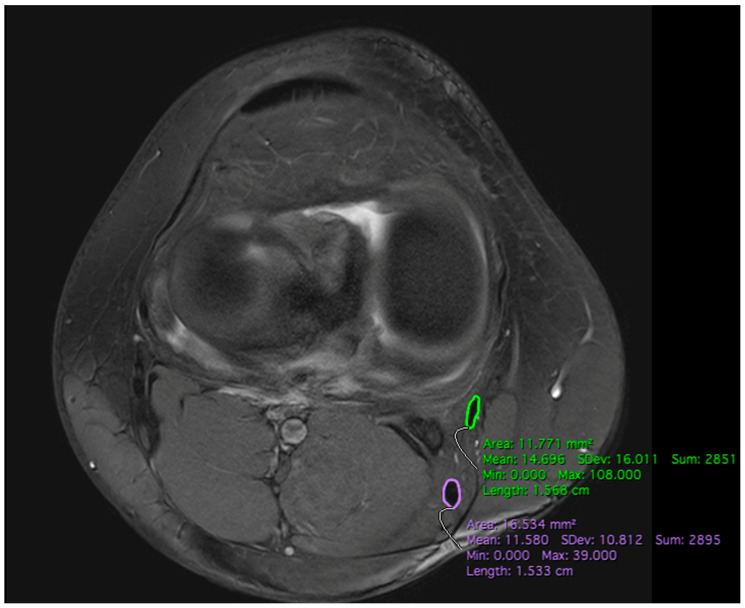
Measurement of the cross-sectional area of the ST (purple) and GT (green) on the level of the joint line using manually drawn closed-polygon function in Horos.

The CSA of the semitendinosus tendon (CSA ST) and gracilis tendon (CSA GT) were each determined by three observers (senior physician, consultant, and medical student) on all six axial slices using the “closed polygon” function in the Horos program (Figure 3). All three observers were trained in detail about the study protocol before the manual tendon sequencing. In 27 subjects, axial T2 sequences were used after determining the different heights in the sagittal plane. Because of the variability of the available MRI datasets, five measurements were performed in axial T1 sequences. For the outline of the two tendons, the images were magnified as much as needed. Afterward, the CSA of the semitendinosus and gracilis tendon on the individual levels were summed up (CSA STGT).

### 2.3. Intraoperative Measurements

Initially, the semitendinosus and gracilis tendons were harvested using a tendon stripper to detach them on the level of the musculotendinous transition. The fibers of the semitendinosus tendon that were pulling toward the medial head of the gastrocnemius muscle were detached prior to this. After the removal of the remaining muscle tissue, the two tendons were then each bundled twice. The resulting four-stranded graft (STGT graft) was then sewn together with a baseball-stitch at both ends over a length of 3 cm each. The diameter of this STGT graft was measured using a graft sizing block (Arthrex Graft Sizing Block AR-1886, Arthrex, Munich, Germany) with holes of 0.5-mm increments. This was determined by the smallest hole through which the graft could pass at its widest diameter with a tight fit but free passage.

### 2.4. Statistical Analysis

For the statistical analysis, IBM SPSS (Statistics for Macintosh, Version 26.0, IBM Corp. Armonk, NY, USA: IBM Corp.) was used. The intraclass correlation coefficient (ICC) was calculated using Cronbach’s alpha to investigate the interrater reliability between the measurements of the three observers. In order to identify a possible correlation between the intraoperative graft diameter and the CSA on the different levels, the Pearson correlation coefficient was calculated. A *p*-value < 0.05 was considered significant. The Kolmogorov–Smirnov test was used to check whether the data were normally distributed.

## 3. Results

The complete datasets of 32 patients with preoperative MRI sequencing and intraoperatively documented graft size could be analyzed.

### 3.1. Interrater Reliability

The ICC was categorized as described by Koo et al. [17]. In general, the interrater reliability between the MRI measurements of the three observers was good (ICC > 0.75) to excellent (ICC > 0.9), depending on the investigated level (Table 2). For further correlation of the CSA STGT with the intraoperative four-stranded STGT graft diameter, the measurements of the doctoral student were used.

### 3.2. MRI CSA STGT

The mean CSA STGT on different levels is displayed in Table 3. At the level 20 mm under the JL, the CSA STGT could not be determined in three subjects during incomplete mapping.

### 3.3. Correlation CSA STGT with Intraoperative Graft Diameter

The mean intraoperative four-stranded STGT graft diameter was 8.1 mm ± 0.5 mm (range: 7 mm to 9 mm). The strongest correlation between CSA STGT and the intraoperative STGT graft was found on the level 10 mm above the JL. Additionally, at the level of the joint line and 10 mm under the JL, a strong correlation was observed (Table 4).

## 4. Discussion

The main finding of the present study is that there was a good correlation of the preoperatively measured CSA with the intraoperatively measured quadruple STGT graft, which was strongest at a level of 10 mm above the joint line. Furthermore, there was a negligible interrater variability between examiners with different levels of expertise.

The preoperative evaluation of whether a sufficient graft diameter can be achieved during ACL reconstruction using the hamstring tendons enables the orthopedic surgeon to better plan the surgical procedure. High interrater reliability was shown between the individual examiners regardless of medical experience. Thus, the level of training of the individual examiner appears to have very little influence on the correct segmentation of the two tendons in MRI. In practice, therefore, the measurement of the CSA does not necessarily have to be performed by an experienced senior physician but can be carried out by a trained medical assistant. In previous studies, the CSA was only measured at single levels assessed by using various diagnostic methods to investigate a possible correlation with the intraoperative graft diameter [14,15]. In most cases, the CSA was measured at the level of the WID or the widest point of the medial femoral epicondyle [16]. In comparison with the CSA values of previous studies, the results of the present study showed reproducible results at the corresponding levels [10,18,19,20]. In the study by Beyzadeoglu et al., the investigators used the joint line as the reference level for CSA determination [21]. This also showed comparable results to the present study. However, the degree of correlation between the CSA of the semitendinosus and gracilis tendon with the intraoperative graft diameter showed a very wide range depending on the particular study. Erquicia et al. determined the CSA of the two tendons at the level of the widest point of the medial femoral epicondyle using a standardized MRI protocol and always the same 1.5 Tesla scanner for each MRI. Using 4× magnification, there was a very strong correlation of r = 0.86, *p* < 0.001 of the summarized CSA STGT with the intraoperative graft diameter [10]. Corey et al. determined the CSA at two levels in MRI scans from different institutions without a standardized examination protocol and magnification. At the level at which the examiner considered the two tendons to be largest and roundest (Zakko-method), there was a weak correlation with the four-stranded graft diameter of r = 0.388. Using the level of the WID, the correlation increased to r = 0.427 [19,22]. Hollnagel et al. measured the CSA of the two tendons in images from two MRI devices (1.5 and 3 Tesla) depending on availability. The CSA was examined under maximum magnification at the level of the widest point of the medial femoral condyle and at the joint line. The correlation of the CSA of the single tendons (ST or GT), the sum of the CSA of both tendons, and the mean CSA of both tendons with the intraoperative four-stranded graft diameter was investigated. The strongest correlation was found using the 1.5 Tesla MRI scanner and the sum of the CSA of both tendons (CSA STGT) on the level of the joint line r = 0.703 [23]. The different focuses of the individual studies show which variables can have an influence on the correlation. Thus, in summary, the use of a single MR scanner with standardized sequences and analytical method appears to have a positive effect on the degree of correlation between the CSA of the hamstring tendons with the intraoperative graft diameter. In the present study, images from different MR scanners were included to determine the CSA. This approach realistically represents everyday clinical practice. The innovative character of this study is the measurement of the CSA at several defined levels based on the joint line and the WID. The aim was to determine the level at which the CSA of the two tendons correlated most strongly with the intraoperative graft diameter to generate a recommendation for the best patient individual surgical approach. The results of the present study showed the strongest correlation at the level of the joint line as well as 10 mm above and below it. Increasing the distance from the joint line in the cranial or caudal direction showed a decreasing correlation, which may be due to anatomical conditions. Distally, the tendons changed their shape from round to flat in the area of attachment to the pes anserinus superficialis. This makes it difficult to differentiate the tendon tissue from the surrounding structures on the MRI and makes the measurements more susceptible to segmentation errors. The interpretation of our results suggests that the CSA at the level of the WID, as used in many previous studies, does not seem to be a good predictive parameter to determine the graft diameter. Rather, in clinical practice, the focus should be on the CSA in close vicinity of the joint line to estimate the intraoperative graft diameter.

### Limitations

This study has several limitations. The study population was limited to 32 patients and thus could show divergent results for larger study cohorts and should be considered exploratory. MRI datasets from many different scanner systems were analyzed, which in turn showed quantitative and qualitative variability. Nevertheless, we consider this methodology adequate due to its representation of everyday clinical practice. To generate a robust way to determine intraoperative graft size, we focused on the preoperative CSA of the graft tendons in the present study. Tendon length was not determined in the present setting. This can also help to estimate preoperatively whether a single graft is sufficient or if a STGT is needed.

## 5. Conclusions

The measurement of the CSA STGT in the preoperative MRI enabled a good assessment of the intraoperative achievable graft diameter in ACL reconstruction, independent of the examiners’ training level. The present study suggests that the measurement should be taken in close proximity to the joint line, ideally 10 mm above it, to allow for a reliable estimation of the graft diameter. Patients in whom the adequate graft diameter of a single tendon graft cannot already be evaluated preoperatively should be informed about harvesting both ST and GT.

## Figures and Tables

**Table 1:** Distribution of the anthropometric data of 32 subjects included in the study.

	Mean	SD	Minimum	Maximum
Height (cm)	174.1	±10.9	150.0	192.0
Weight (kg)	74.8	±10.5	52.0	92.0
BMI (kg/cm^2^)	24.7	±3.1	18.8	32.9

**Table 2:** Interrater reliability ^1^.

Level	CSA ST	CSA GT	CSA STGT
20 mm	0.95	0.94	0.95
10 mm	0.96	0.93	0.96
JL	0.84	0.95	0.92
−10 mm	0.95	0.91	0.95
−20 mm	0.89	0.60	0.85
WID	0.92	0.87	0.94

^1^ Values are expressed as the intraclass correlation coefficient for the three investigators using Cronbach’s alpha.

**Table 3:** Cross-sectional area STGT ^1^.

Level	Mean	SD	Minimum	Maximum
20 mm	19.58	±4.26	13.12	28.74
10 mm	19.92	±4.33	13.40	28.64
JL	20.97	±5.26	10.76	31.85
−10 mm	18.84	±4.62	7.15	28.37
−20 mm	16.60	±5.30	6.95	27.85
WID	20.45	±4.32	13.38	30.09

^1^ Values are expressed as the sum of the cross-sectional areas of ST and GT (STGT) in mm^2^.

**Table 4:** Correlation ^1^.

Level	Correlation Coefficient r	*p*-Value
20 mm	0.367	0.019 *
10 mm	0.489	0.002 *
JL	0.438	0.006 *
−10 mm	0.454	0.005 *
−20 mm	0.314	0.049 *
WID	0.410	0.010 *

^1^ Values are expressed as the Pearson correlation coefficient r between the intraoperatively determined four-stranded graft diameter and cross-sectional area STGT with the level of significance *p*. Significant values are marked with *.

## Data Availability

All data are within the manuscript.
